# Supplementary material for: LKB1 Differently Regulates Adipogenesis in Intramuscular and Subcutaneous Adipocytes through Metabolic and Cytokine-Related Signaling Pathways
Source: Cells. 2020 Dec 4;9(12):2599. doi: 10.3390/cells9122599 (PMC7761942; doi:10.3390/cells9122599)
Supplement: Supplementary file 1 [file cells-09-02599-s001.pdf]

LKB1 differently regulates adipogenesis in intramuscular and subcutaneous adipocytes through metabolic and cytokine-related signaling pathways

Supplementary materials

Supplementary Figure 1

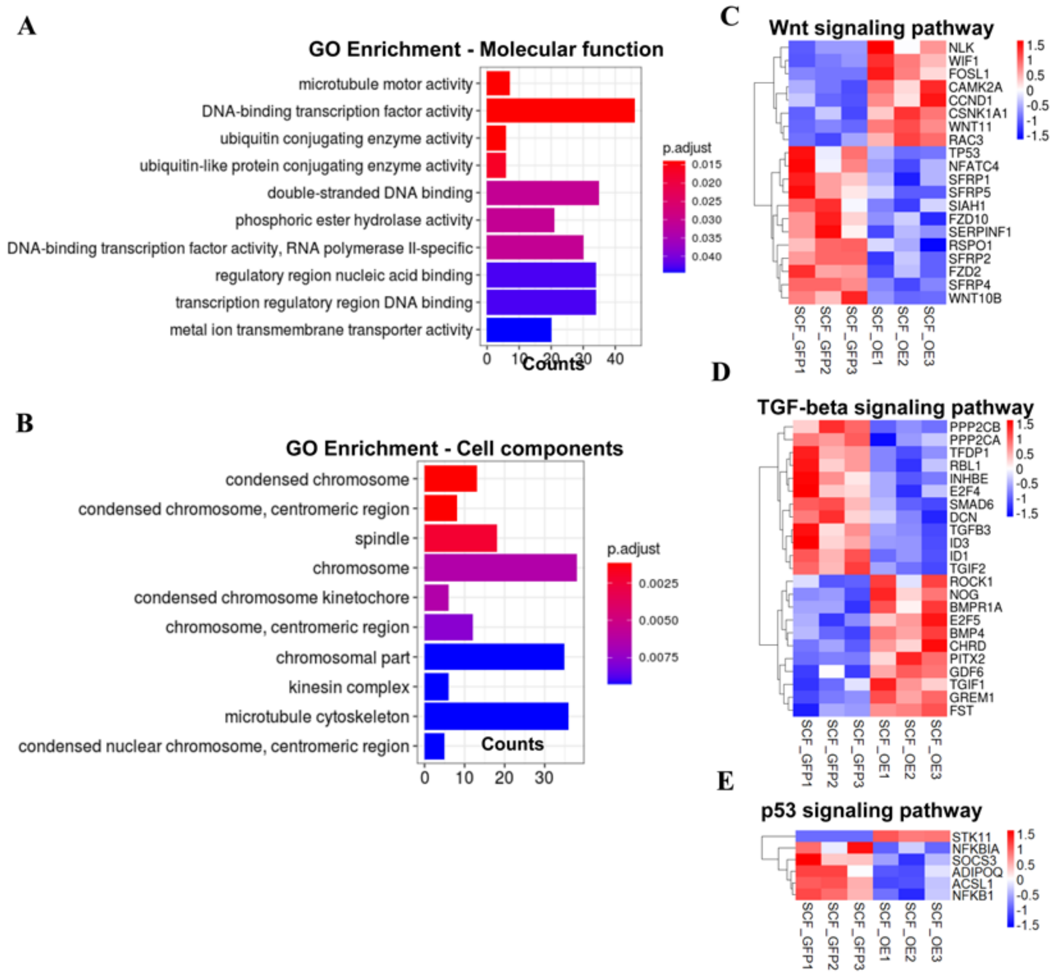

Supplementary Figure 1. LKB1 overexpression alters the transcriptional profile of SCF adipocytes (**A, B**) Gene Ontology (GO) enrichment analysis based on molecular function (**A**) and cell components (**B**) and enriched terms were visualized by bar plot. The bar color indicates significances and the corresponding significance values are displayed as P.adjust. The bar length indicates significantly changed gene counts of genes involved in certain categories. (**C-E**) Heatmap of TPM expression values genes involved in the Wnt signaling pathway (**C**), TGF-beta signaling pathway (**D**) and p53 signaling pathway (**E**). Only genes with $P < 0.05$ are displayed.

Supplementary Figure 2

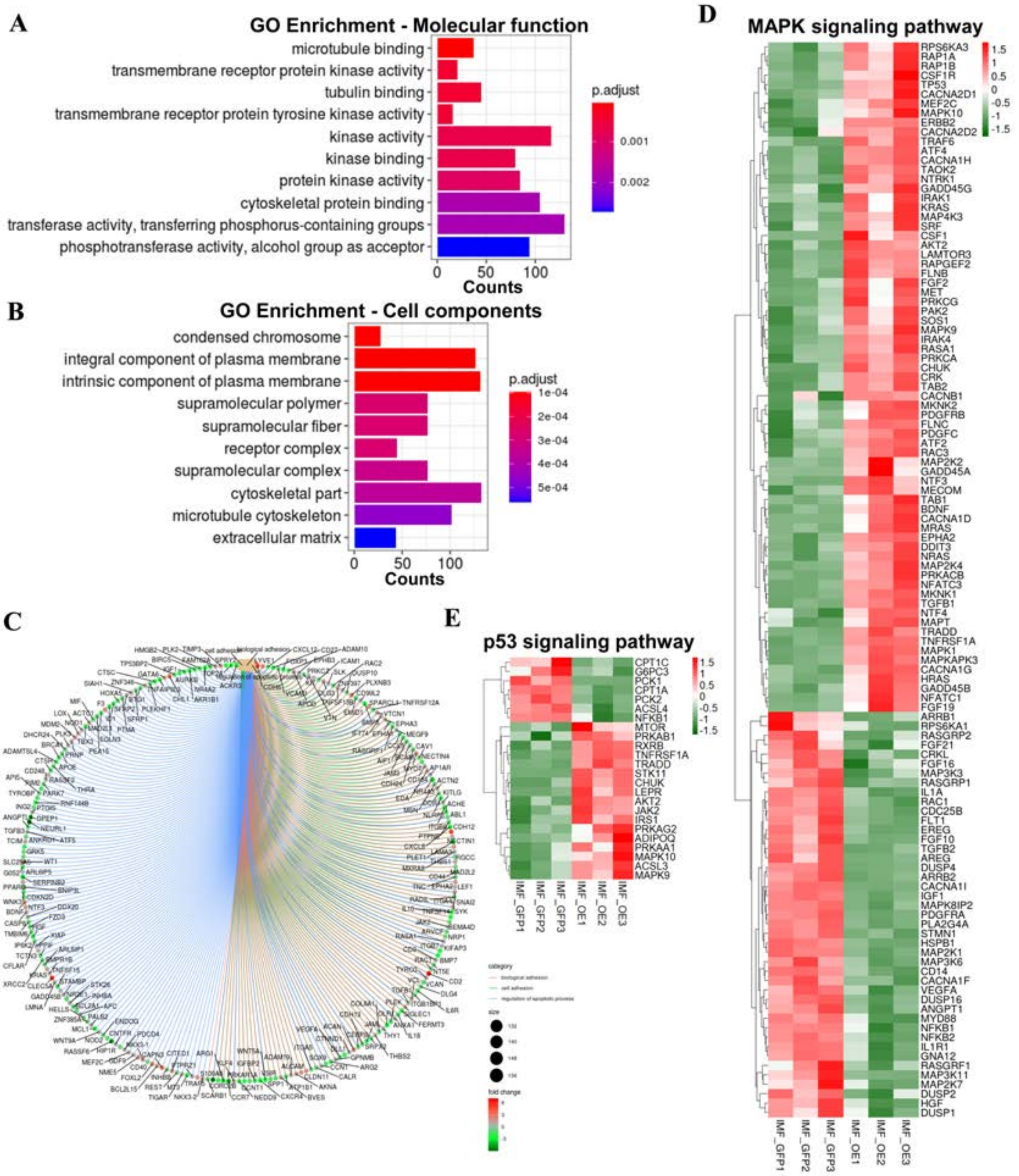

Supplementary Figure 2. LKB1 overexpression alters the transcriptional profile of IMF adipocytes. (**A, B**) Gene Ontology (GO) enrichment analysis based on molecular function **(A)** and cell components **(B)** and enriched terms were visualized by bar plot. The bar color indicates significance, and the corresponding significance values are displayed as P.adjust. The bar length indicates significantly changed gene counts of genes involved in certain categories. (**C**) The cnetplot depicted the linkages of the three most enriched GO terms (cell adhesion, biological adhesion, regulation of apoptotic process) and genes involved in these terms as a network. The yellow dots indicate enriched GO terms and the size of each dots indicates gene counts involved in certain GO terms. These smaller dots indicate genes involved in these terms. The color of each smaller dot indicates log2(fold change) values genes in LKB1-overexpressing IMF adipocytes versus controls. (**D, E**) Heatmap of TPM expression values genes involved

in the MAPK signaling pathway (**D**) and p53 signaling pathway (**E**). Only genes with P < 0.05 are displayed.

Supplementary Figure 3

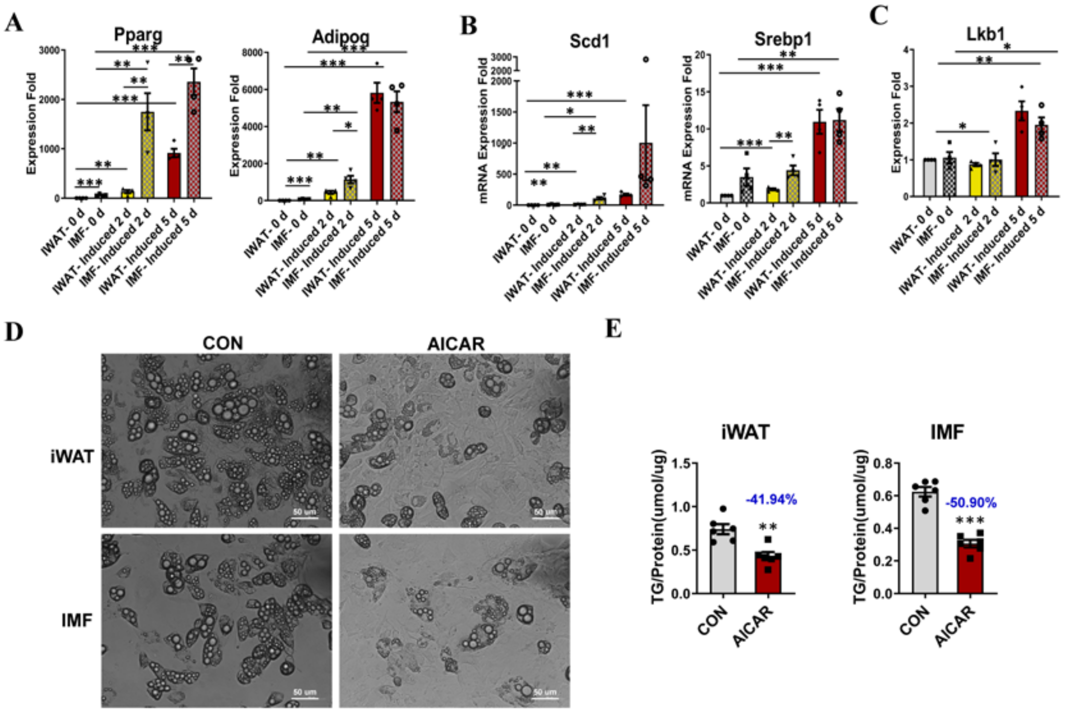

Supplementary Figure 3. Expression levels of adipogenesis-related genes in mouse IMF and iWAT adipocytes during adipogenic differentiation. (**A-C**) q-PCR results of the mRNA levels of *Pparg*, *Adipoq* (**A**), *Scd1*, *Srebp1* (**B**), *Lkb1* (**C**) in iWAT and IMF adipocytes before and after adipogenic differentiation induction (n = 4). (**D**) Optical microscope images of differentiated iWAT and IMF adipocytes exposed to AICAR (1MM) or control. (**E**) TG contents of differentiated iWAT and IMF adipocytes exposed to AICAR (1MM) or control. Data is presented as the means ± SEM. * P < 0.05, *** P < 0.001.

Supplementary Table 1. Primers used for qPCR.

Gene	Primer Name	Primer Sequence (5′-3′)
m-*Lkb1*	Forward	TTGGGCCTTTTCTCCGAGG
	Reversed	CAGGTCCCCCATCAGGTACT
m-Cebpa	Forward	TTACAACAGGCCAGGTTTCC
	Reversed	GGCTGGCGACATACAGTACA
m-Adipoq	Forward	TGTTCCTCTTAATCCTGCCCA
	Reversed	CCAACCTGCACAAGTTCCCTT
m-Scd1	Forward	CGCTGGCACATCAACTTCAC
	Reversed	AGGAACTCAGAAGCCCAAAGC
m-Srebp	Forward	GGAGCCATGGATTGCACATT
	Reversed	GGCCCGGGAAGTCACTGT
m-Pparg	Forward	TCGCTGATGCACTGCCTATG
	Reversed	GAGAGGTCCACAGAGCTGATT

m-Cpt1a	Forward	TGTCCAAGTATCTGGCAGTCG
	Reversed	CATAGCCGTCATCAGCAACC
m-Il1b	Forward	GCAACTGTTCCTGAACTCAACT
	Reversed	ATCTTTTGGGGTCCGTCAACT
m-Ccl7	Forward	GCTGCTTTCAGCATCCAAGTG
	Reversed	CCAGGGACACCGACTACTG
m-Ucp1	Forward	AGGCTTCCAGTACCATTAGGT
	Reversed	CTGAGTGAGGCAAAGCTGATTT
m-Pgc1a	Forward	TATGGAGTGACATAGAGTGTGT
	Reversed	CCACTTCAATCCACCCAGAAAG
m-Prdm16	Forward	CCACCAGCGAGGACTTCAC
	Reversed	GGAGGACTCTCGTAGCTCGAA
m-Cox5a	Forward	TTGATGCCTGGGAATTGCGTAAAG
	Reversed	AACAACCTCCAAGATGCGAACAG
m-Cox7a	Forward	GCTCTGGTCCGGTCTTTTAGC
	Reversed	GTACTGGGAGGTCATTGTCGG
m-18s	Forward	AGTCCCTGCCCTTTGTACACA
	Reversed	CGATCCGAGGGCCTCACTA
p-Pparg	Forward	TGTGGACCTGTCGGTGATG
	Reversed	TGGAGTGGAAATGCTGGAGA
p-Cebpa	Forward	CGGTGCGTCTAAGATGAGG
	Reversed	AGCGGTGAGTTTGCGTTT
p-Adipoq	Forward	CTGGCGAGAAGAGTGAGA
	Reversed	TGCTGAACGGTAGACATAGGC
p-Fabp4	Forward	GGAAGGTGGCTGGCATGGC
	Reversed	CCTCCATCTAAGGTTATGGTGCTCTTG
p-Leptin	Forward	AGATCCTCACCAGTCTGCCTTCC
	Reversed	CCAGGCTCTCCAAGGTCTCCAG
p-Lkb1	Forward	TTTACAGAGGGCGAGCTGAT
	Reversed	CATCAGGTACTTGCCGATGA
p-Hsl	Forward	ACCCTCGGCTGTCAACTTCTT
	Reversed	ACTTTCTCCTCCTTGGTGCTAATCT
p-Atgl	Forward	GCACATCTCTCGAAGCACCA
	Reversed	GCACATCTCTCGAAGCACCA
p-Glut4	Forward	GAAGGAAGAAGGCAATGCTG
	Reversed	GAGGAACCGTCCAAGAATGA
p-18s	Forward	CCCACGGAATCGAGAAAGAG
	Reversed	TTGACGGAAGGGCACCA
